# Supporting play exploration and early developmental intervention versus usual care to enhance development outcomes during the transition from the neonatal intensive care unit to home: a pilot randomized controlled trial

**DOI:** 10.1186/s12887-018-1011-4

**Published:** 2018-02-09

**Authors:** Stacey C. Dusing, Tanya Tripathi, Emily C. Marcinowski, Leroy R. Thacker, Lisa F. Brown, Karen D. Hendricks-Muñoz

**Affiliations:** 1 Department of Physical Therapy, Motor Development Lab, Virginia Commonwealth University, Office: 1200 E Broad St. B106, PO BOX 980224, Richmond, VA 23298 USA; 2 Rehabilitation and Movement Sciences Program, Virginia Commonwealth University, Richmond, USA; 3 Schools of Nursing and Medicine, Virginia Commonwealth University, Richmond, USA; 4 School of Nursing, Virginia Commonwealth University, Richmond, USA; 5 Children’s Hospital of Richmond, Virginia Commonwealth University, Richmond, USA

## Abstract

**Background:**

While therapy services may start in the Neonatal Intensive Care Unit (NICU) there is often a gap in therapy after discharge. Supporting Play Exploration and Early Development Intervention (SPEEDI) supports parents, helping them build capacity to provide developmentally supportive opportunities starting in the NICU and continuing at home. The purpose of this single blinded randomized pilot clinical trial was to evaluate the initial efficacy of SPEEDI to improve early reaching and exploratory problem solving behaviors.

**Methods:**

Fourteen infants born very preterm or with neonatal brain injury were randomly assigned to SPEEDI or Usual Care. The SPEEDI group participated in 5 collaborative parent, therapist, and infant interventions sessions in the NICU (Phase 1) and 5 at home (Phase 2). Parents provided daily opportunities designed to support the infants emerging motor control and exploratory behaviors. Primary outcome measures were assessed at the end of the intervention, 1 and 3 months after the intervention ended. Reaching was assessed with the infant supported in an infant chair using four 30 s trials. The Early Problem Solving Indicator was used to evaluate the frequency of behaviors during standardized play based assessment. Effect sizes are including for secondary outcomes including the Test of Infant Motor Performance and Bayley Scales of Infant and Toddler Development.

**Results:**

No group differences were found in the duration of toy contact. There was a significant group effect on (F1,8 = 4.04, *p* = 0.08) early exploratory problem-solving behaviors with infants in the SPEEDI group demonstrating greater exploration with effect sizes of 1.3, 0.6, and 0.9 at the end of the intervention, 1 and 3 months post-intervention.

**Conclusions:**

While further research is needed, this initial efficacy study showed promising results for the ability of SPEEDI to impact early problem solving behaviors at the end of intervention and at least 3 months after the intervention is over. While reaching did not show group differences, a ceiling effect may have contributed to this finding. This single blinded pilot RCT was registered prior to subject enrollment on 5/27/14 at ClinicalTrials.Gov with number NCT02153736.

**Electronic supplementary material:**

The online version of this article (10.1186/s12887-018-1011-4) contains supplementary material, which is available to authorized users.

## Background

In the United States 1 in 8 infants are born prematurely (< 37 weeks gestation), placing the infants at increased risk for learning difficulties, lower quality of life, and motor disabilities with up to 50% of infant born very preterm requiring special education [1, 2]. Infants born preterm with neonatal white matter injury are also at higher risk of having cerebral palsy (CP), cognitive impairments, requiring more teacher attention, and having an increased need for special education support [3–6]. While survival of infants born preterm is more certain than ever, developmental services typically use a “wait and see” approach to start intervention and once enrolled provides low intensity intervention resulting in little to no lasting effects on motor and cognitive development [7, 8]. Basic science and clinical evidence suggest early and intense intervention is more effective than a long-term low intensity approach at promoting neural recovery in adults and children as well as in animal models of cerebral palsy [9–12]. Evidence-based, effective early intervention programs are needed to target early motor abilities that support motor and cognitive development in infants at high risk of having cerebral palsy or minor neurological dysfunctions.

Motor and cognitive development are tightly coupled, suggesting that delays in one domain could contribute to delays in other domains [13–17]. Motor experience provides infants an opportunity to learn about objects and interaction supports development in multiple domains [17–20]. The action perception model of development is governed by the theory that motor activity contributes to the infants attempts to attend to the environment, allowing the infant to receive and interpret important information, and solve problems by linking the mind and body in a cycle that supports development [21]. Children with motor impairments or delays have limited ability to interact with and interpret the environment, restricting their opportunities to learn through action [16]. Atypical postural control and impaired reaching abilities are common in infants born preterm and infants later diagnosed with development deficits such as CP, developmental coordination disorder, and minor neurological dysfunction [22–27]. Children born preterm with motor coordination disorders or CP score lower on problem-solving tasks than those without motor disabilities at school age [14, 15]. The relationship between motor and cognitive outcomes in infants born preterm supports the need for interventions that incorporate both the motor and cognitive domains and the interaction between these domains to maximize outcomes.

Developmental interventions for infants born preterm often focus on one approach; motor, cognitive, or parent-children interactions. A recent Cochrane review demonstrated that intervention to support motor development were slightly more effective when initiated in the NICU, and was more effective when intervention strived to impact both parent-child interaction and infant development [28].

The purpose of this study was to assess the initial efficacy of Supporting Play, Exploration, and Early Developmental Intervention (SPEEDI) an intervention that started in the NICU and continued for 12 weeks in the community. The goals of SPEEDI were to provide an enriched environment and increased opportunities for infant initiated movements through collaborative parent, therapist and infant interactions during the first months of life in order to enhance the infant’s development during and after the intervention period (Additional file 1).

Therefore, the primary aims of this single blinded randomized controlled trial were to evaluate the short-term efficacy of SPEEDI at enhancing reaching and play based exploratory problem solving compared to infants receiving usual care. We hypothesized that compared to the usual care group, the SPEEDI intervention group would demonstrate increased reaching and early problem solving skills at the end of the intervention, 1 and 3 months after the intervention ended. The secondary aims were to explore the impact of SPEEDI on longer-term motor and cognitive development.

## Methods

### Design overview

This study is a single blinded randomized pilot clinical trial.

### Setting and participants

Every infant admitted to a single level IV NICU during the enrollment period was screened for eligibility. Infants born extremely preterm (<29 weeks of gestation) and/or with neonatal diagnosis of a brain injury, who lived within 30 min of the hospital, and spoke English were eligible for this study. Brain injuries included intraventricular hemorrhage (grade 3 or 4), periventricular white matter injury, hypoxic ischemic encephalopathy or hydrocephalus requiring a shunt. Exclusion criteria included: a diagnosis of a genetic syndrome (e.g., Trisomy 21) or musculoskeletal deformity. (e.g., limb deficiency). Information was provided to parents of eligible infants between 35 and 40 weeks of gestation if the infant was off ventilator support by 40 weeks of gestation. Only one infant from eligible multiple births was enrolled in the study. The infant’s medical records were used to document medical complications and score the Neonatal Medical Index [29]. All infants received a packet of age-appropriate infant toys and total of $100 to offset travel, parking, and time meeting with the study staff.

### Randomization and interventions

Infants were randomized to the intervention or usual care group after a baseline assessment using a stratified (brain injury / no brain injury) randomization scheme. All infants, regardless of group assignment, participated in usual care as it was deemed unethical to withhold routine care. Usual care included referral to therapy services in the NICU at the medical team discretion and referral to their local Early Intervention (EI) program. EI was provided in accordance with state implementation guidelines under the United States Individuals with Disabilities Education Improvement Act (IDEIA) [30]. Parents were offered referral by NICU staff prior to discharge, during visits to the Neonatal Continuing Care Program, or by the study assessment team if requested by a parent. In order to document the usual care services provided outside of the study protocol, NICU medical records were reviewed and parents were asked to fill out a questionnaire at each assessment visit to document enrollment in and frequency of outpatient or EI therapy visits.

Infants enrolled in the usual care group received only usual care in the NICU and community. Infants enrolled in the SPEEDI group participated in a 2 phase intervention utilizing principles of the synactive theory of development and action perception theory to train parents to provide daily intervention to support the infant’s development through environmental enrichment and active engagement (Additional file 1). The first phase, delivered face to face in the NICU, focused on helping parents identify ideal times to interact with their infant, provide developmentally appropriate interaction and start to consider how they will interact with their infant after NICU discharge [31, 32]. All sessions were designed to include some time with the infant, discussion of behavioral cues and development, and answering the parents’ questions. Videos were provided for parents to review between sessions (Additional file 2). An activity booklet was reviewed with the parent during the last few visits in phase 1 in preparation for phase 2 (Additional file 3). Phase 2 focused on parents using the skill acquired during phase 1 to provide their infant with daily opportunities for motor and problem-solving based play with a goal of improving motor skills and early problem solving (Table 1). The Phase 2 intervention is based on action-perception theory which stresses the important role of early experience in shaping development [13]. SPEEDI applies this theory by engaging parents in providing early experiences that are the “just right challenge” for the infant that day, matching the demand with the infant’s ability to support ongoing development. A focus was placed on allowing the infant to use self-directed movements, variability in movement pattern, and active infant engagement through parental support and environmental enrichment (Additional file 1 Key Principles). Parents were encouraged to progress the activities from easier (stage 1) to harder (stage 2) activities over the 12 weeks of phase 2 intervention. Study interventionists meet with parents in their home 5 times during phase 2 to support the parents’ abilities to progress the intervention. Parents were encouraged to contact their interventionist with any questions or concerns between visits. The visit schedule was flexible to meet the family’s needs, but the same number of visits were provided for all infants.

**Table 1:** SPEEDI Intervention Description

Phase 1 (21 days starting when medically stable)	Phase 2 (12 weeks starting at the end of phase 1)
In NICU	Primarily at home, but started in NICU if not ready for discharge on day 21 post baseline.
5 intervention sessions provided by the parent and therapist jointly and in response to the infant’s behavioral cues based on the synactive theory of development [31, 32].	Parents were encouraged to provided activities daily, with a goal of at least 20 min per day of activities 5 days per week, provided by the parent
33 Videos of positive and negative interaction available to parents throughout the phase 1 intervention as examples (Additional file 2)	An activity booklet (Additional file 3), with pictures, simple text, and a log for parent to record daily activities and questions was used to encourage parents to provide motor and cognitive opportunities daily in a variety of play positions, environments, and with objects [13].
Coaching on behavioral states, self-calming, environmental modification, and choosing times for feeding and play based interactions using dolls or video clips if the infant was not alert or fatigued	Parent encourage to provide the “just right challenge” advancing from stage 1 to stage 2 activities as they observed their infant improving or discuss with therapist at each visit
Provide experience with variable and self-directed movements and social interaction without physiological or behavioral stress. Introduced phase 2 activities by end of phase1	Physical Therapist participated in 5 parent-infant activity session over 12 weeks and helped with advancing from stage 1 to stage 2 activities as the infant was ready.
Guided participation used in identifying cues to stop, alter, or delay interactions during caregiving, feeding, play activities	Parent was encouraged to develop a daily routine for encouraging developmental play.

Over arching theme: Encouraging parents to provide the “just right challenge” by pacing intervention and the experiences provided based on the infant’s behavioral state, signs of stress including autonomic, motor, or attention changes and demonstrated readiness for increasing duration or difficulty of developmental play skills

Key principles: Encourage self-initiated movement, variability, object interaction, and social interaction. Do not impose movement on the infant. Observe and respond to the infant’s behavioral cues. (Additional file 1)

Key Strategies to support motor development during interactions: provide graded postural support, observe spontaneous movement in response to your support, vary postural support to encourage different opportunities and sensory input, vary positioned with the minimal support to encourage variable movements

The study interventionists were both board certified pediatric physical therapist with extensive experience providing intervention in the NICU and in the first months of life. They were trained using a detailed manual, having previously participated in a feasibility study of SPEEDI, and met at regular intervals to discuss intervention strategies. To ensure ongoing adherence to the key principles of the intervention, the interventionists: 1) completed a fidelity checklist self-reflecting on whether they had covered the key intervention principles and used key intervention strategies (Table 1) after each phase 1 visits, 2) reflected on the parents’ use of the key principle and strategies during the collaborative sessions in phase 2, and 3) 30% of the intervention sessions were video recorded and fidelity scored by the other interventionist. In order to track adherence and approximate dose of intervention provided by parents the data from the daily activity log within the activity booklet was used to compare anticipated with actual days of intervention and progression from stage 1 to stage 2 activities (Additional file 3).

### Outcomes and follow up

All infants enrolled in the study were assessed on the same schedule by a physical therapist blind to group assignment who completed extensive training and reached reliability on all outcome measures prior to the study. The assessment schedule was baseline, End phase 1 (21 days after baseline), End phase 2 (12 weeks after End Phase 1), Follow up 1 (1 month after End phase 1, and Follow up 2 (2 months after follow up 1 or 3 months after End phase 2) (Table 2). A priori power analysis using data from a feasibility study determined a sample size of 14 infants, 7 per group, was needed to detect group differenced on the primary outcomes with alpha 0.10 and 80% power [33]. The secondary outcomes were included in the protocol to allow for further analysis and the estimation of effect sizes for future research.

**Table 2:** Assessment schedule

Domain	Baseline	End Phase 1	End Phase 2	Follow up 1	Follow up 2	12 months Adjusted Age
	Day 0	Day 21	Day 111 (15 weeks)	Day 141 (20 weeks)	Day 201 (29 weeks)	Target Day 382 –clinical visit
Therapy or EI Services	EMR	EMR	Parent survey	Parent survey	Parent survey	Clinical records
Seated Exploration and Reaching			Hands midline and Reaching	Hands midline and Reaching	Hands midline and Reaching	
Problem Solving			EPSI	EPSI	EPSI	
Motor	TIMP	TIMP	TIMP	TIMP	Bayley	Bayley
Cognition					Bayley	Bayley
Language					Bayley	Bayley

### Primary outcomes

#### Reaching skill

Reaching was assessed at end phase 2 and both follow up visits. The infant was positioned in an infant seat that provided trunk support and was reclined to 20 degrees while two synchronized video cameras placed at 45 degrees on the left and right sides were used to record the anteriolateral views of the infant’s behavior. Reaching skill was assessed using four 30 s trials. An infant rattle was presented to the midline of the infant’s chest at 75% of the infant’s arm length. An additional eleven trials were presented under 3 conditions to explore early arm use, however these results are not presented in this manuscript as they were not directly related to the primary or secondary aims. Behavioral coders marked each time the infants’ hand was in contact with a toy and the duration of each behavior was calculated using behavioral coding software.^1^ A toy contact was coded whenever any portion of the infant’s hand, distal to the wrist, was in contact with a toy, regardless of hand position. The two coders were blind to group assignment. On 20% of visits, reliability was calculated using a percentage agreement at each visit: [agreed/ (agreed + disagreed)] * 100. Intra rater and inter rater agreement for toy contact 95.4 and 97.0, respectively.

#### Exploratory problem-solving behaviors

Problem-solving behaviors were assessed using the Early Problem Solving Indicator (EPSI) at end phase 2 and both follow up visits. The EPSI is the cognitive subtest of the Individual Growth and Development Indicators designed to measure infant and toddler play-based problem-solving from 6 to 36 months of age. While the infants in this study were initially less than 6 months of age, the final study visit was at about 6 months of age and two of the four behaviors coded as part of the EPSI are commonly observed in young infants. So this tool was deemed the best available to document early-problem solving behaviors during play. The EPSI defines problem-solving as consisting of visual exploration, object manipulation and memory [34]. Previous studies with the EPSI show adequate reliability and validity, and usefulness in documenting change over time [35, 36]. During the EPSI, the infant was video-recorded interacting with 3 standard toys: pop-up animals toy, 6 seriated plastic cups, and a pound a ball game with a hammer and 4 balls. Infants were given each toy for 2 min while the examiner supported the child in sitting (pop up and cups) and prone (pound a ball) in order to sample 2 common play positions. If needed the examiner used a standard set of prompts such as tapping on the toy at a consistent frequency to engage or re-engage the infant in the standardized toy without demonstrating the use of the toy. The lead author has been certified by the EPSI developer to train blinded examiners and coders.

The frequency of 4 behaviors (look, explore, function, solution) were coded using definitions from the EPSI protocol. These behaviors were mutually exclusive, so only one behavior is coded at any time. Look was coded when the infant was looking at the toy. Explore was coded when the infant touched, manipulated, mouthed, rubbed, shook, pushed, pulled, banged, threw, or dropped the toy. A function was coded if the infants used the toy in a manner in which it was intended but does not require that the child complete all of the functions of the toy (e.g., moved one lever to make an animal pop up or nesting any 2 cups). A solution was coded if the infant used the toy in a way that its full functionality was displayed (e.g., moved all levers and buttons, so that all animals popped up or nesting all the cups in order). Two coders who were blinded to the infant’s group assignment, recoded 20% of visits, including some from each of the 4 study visits, with an inter rater agreement of 94.0% and intra rater agreement of 97.7%. The total number of problem solving behaviors was calculated as a sum of look, explore, function, and solution for each infant at each visit to represent that infant’s problem solving abilities.

### Secondary outcome measures

#### Neuromotor control and development

The Test of Infant Motor Performance (TIMP) and Bayley Scales of Infant and Toddler Development, third edition (Bayley) were included, because they are commonly used clinical assessments in the population and ages included in this study. The TIMP was administered at the baseline, end of each phase of intervention, and at the first follow-up visit. TIMP raw score can ranges from 0 to 142. The Bayley was administered at the final follow-up visit and 3 months after the intervention ended [37, 38]. Normative values on the Bayley include Composite Scores for Cognitive, Language, and Motor with a mean of 100 and a standard deviation of 15. In order to quantify the longer-term outcomes, Bayley scores from the Neonatal Continuing Care Program at 12 months of adjusted age were extracted from the infant’s medical record if available. All infants in this study meet the criteria for referral to this clinic and appointments were scheduled at NICU discharge. In all but 1 case, the examiner in the clinic was blinded to group assignment at the clinic visit.

### Statistical analysis

Descriptive statistics were used to describe the study sample. The planned sample size and statistical significance was a priori set with an ɑ level of 0.10 level to reduce the risk of missing small, but important group differences in this first efficacy study of SPEEDI (i.e., Type II error). To assess the primary outcomes of Toy contact (reaching) and Frequency of total problem-solving behaviors a repeated measures ANOVA (RMANOVA) [39] was fit using a mixed linear model (MLM). The model fit included a between subjects factor (Group: Intervention, Control), one within subject factor (Time: Assessment time point of end phase 2, follow up 1, and follow up 2) and the interaction between Group and Time. Post-hoc analysis of the types of problem solving behaviors was completed to quantify changes in exploratory problem solving not reflected in the total problem-solving behavior score. Secondary outcome measures were assessed to estimate effect sizes. Effect sizes were calculated using change in TIMP raw score from baseline to end phase 2 and to evaluate group differences on the Bayley Motor, Language, and Cognitive Composites at 3 months post intervention and at 12 months adjusted age. Due to the preliminary nature of this study, no corrections for multiple comparisons were used.

## Results

Fourteen infants meet the inclusion criteria and enrolled. Median birth weight, gestational age, gender, race, ethnicity, and number of infants with a brain injury were similar between groups (Table 3). In the SPEEDI intervention group mothers were significantly younger, infants were sicker (higher NMI scores) and started the study at an older age (Table 3). The majority of mother’s in both the groups reported living in poverty and did not have a college education. The majority of the sample was African American (Table 3).

**Table 3:** Description of subjects

	Total *n* = 14	Control *n* = 7	SPEEDI *n* = 7	*p*-value^e^
Maternal Age^b^	29.50 (27.00, 31.00)	31.00 (29.00, 42.00)	27.00 (23.00, 31.00)	0.05^d^
Maternal Education^a^				0.14^c^
HS or Less	46% (6/13)	33% (2/6)	57% (4/7)	
Some College	23% (3/13)	50% (3/6)	0% (0/7)	
College or More	31% (4/13)	17% (1/6)	43% (3/7)	
Household Income^a^				
< $24,000 (poverty)	50% (7/14)	57% (4/7)	43% (3/7)	1.00^c^
$24,001 - $ 36,000	50% (7/14)	43% (3/7)	57% (4/7)	
> $36,001 (1.5 time poverty)	0% (0/14)	0% (0/7)	0% (0/7)	
Gender Male^a^	57% (8/14)	43% (3/7)	71% (5/7)	0.59^c^
Birth Weight (g)^b^	795.00 (615.00, 1190.00)	840.00 (700.00, 320.00)	680.00 (580.00, 1190.00)	0.48^d^
Gestational Age (wks)^b^	25.50 (25.00, 27.00)	26.00 (25.00, 28.00)	25.00 (24.00, 27.00)	0.44^d^
Race^a^				
Caucasian	14% (2/14)	14% (1/7)	14% (1/7)	
African American	72% (10/14)	72% (5/7)	72% (5/7)	
Biracial	7% (1/14)	0% (0/7)	14% (1/7)	
Other	7% (1/14)	14% (1/7)	0% (0/7)	
Ethnicity^a^				
Hispanic	7% (1/14)	14% (1/7)	0% (0/7)	
Non-Hispanic	93% (13/14)	86% (6/7)	100% (7/7)	
IVH (Any grade)^a^	36% (5/14)	14% (1/7)	57% (4/7)	0.27^b^
IVH Grade 3 or 4^a^	14% (2/14)	14% (1/7)	14% (1/7)	1.00^b^
HIE^a^	7% (1/14)	14% (1/7)	0% (0/7)	1.00^c^
PVL^a^	14% (2/14)	0% (0/7)	29% (2/7)	0.46^b^
Days in NICU^b^	116.50 (93.00, 125.00)	93.00 (65.00, 107.00)	125.00 (116.00, 126.00)	0.14^d^
NMI Rating^b^	5.00 (4.00, 5.00)	5.00 (4.00, 5.00)	5.00 (5.00, 5.00)	0.06^d^
Adjusted Age^b^				
Baseline (weeks of gestation)	38 (35, 39)	35 (35, 39)	39 (36, 40)	0.05^d^
End Phase 1 (weeks of gestation)	40 (38, 42)	38 (38, 41)	42 (40, 43)	0.05^d^
End phase 2 (weeks of adjusted age or beyond 40 weeks of gestational age)	13.5 (11.0, 15.0)	13.0 (11.0, 14.0)	15.0 (13.0, 15.0)	0.28^d^
Follow-up 1 (1 month after intervention)	18.0 (16.0, 19.0)	16.0 (15.0, 18.0)	19.0 (18.0, 20.0)	0.09^d^

Notes: ^a^Percent (n/total)

^b^Median (IQR)

^c^Fisher’s Exact Test

^d^Mann-Whitney U Test (Wilcoxon Rank-sum test)

^e^Between Group Differences Unadjusted for multiple comparisons

**Fig. 1:**
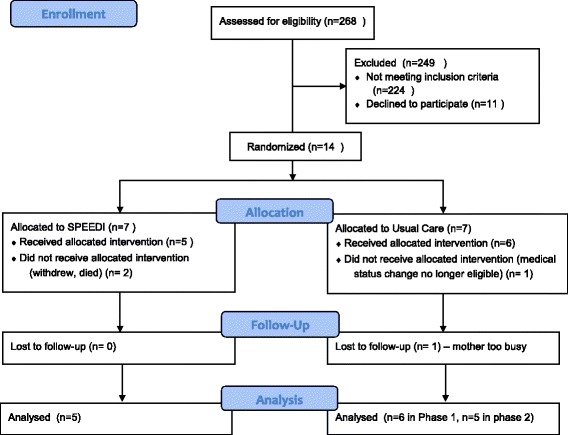
CONSORT Flow chart. This flow chart showing the recruitment and retention of participants in each arm of the clinical trial

A total of 4 infants, 2 in each group and 2 with brain injury, did not complete the study. Three infants were lost while still in the NICU, 1 infant in each group was unable to continue for medical reasons and 1 infant in the intervention group withdrew after the baseline assessment. The data for these 3 infants were excluded from all outcome assessment. One additional infant, from the usual care group, could not be reached for follow up visits after NICU discharge thus only his baseline and end phase 1 data (TIMP only) were included (Fig. 1).

### Description of usual care

Fifty percent of the infants enrolled in the study were receiving therapy services in the NICU at baseline. Infants received a mean of 6.0 visits (range 2–12) from PT and 3.8 visits (range 0–7) from OT during the 21 days of Phase 1 of this study. All infants, except 1 in the SPEEDI group, had been assessed for EI services by follow up 1. Only 4 of the infants were receiving direct therapy services, 3 in the control group, with an average of 1.4 therapy visits per month planned based on parent report.

### Fidelity of SPEEDI intervention

The SPEEDI therapist’s adherence to the key principles of SPEEDI was 87.9% on self report and 86.5% scored by a second rater. All phase 1 sessions were completed with a mean duration of 45 min. During 4 out of 25 sessions, limited infant alertness necessitated discussion and simulation rather than interaction with the infant. The 7 key principle of the intervention were reviewed an average of 3.6 times each during Phase 1. Each of the 4 intervention strategies were used an average of 3.7 times over the 5 sessions and an average of 3.0 strategies were used per sessions.

During Phase 2, infants received all 5 parent/therapist home based intervention sessions with a mean duration of 35 min. During 1 session with 3 different infants, the infant was too sleepy for the parent to demonstrate the SPEEDI intervention activities during the phase 2 sessions. The therapist and parent talked about the parents observations and simulated the activities as needed during these sessions. Parents addressed a mean of 5.6 key principles per session with principles being addressed a mean of 4.0 out of a possible 5 times during phase 2. Each of the 4 key intervention strategies were used an average of 3.9 times with an average of 3.1 key strategies used in each session.

Parent/infant dyads were expected to document 53 days of intervention between the end of phase 1 assessment visit and the last intervention visit. Parent documented a mean of 63.8 session (range 52–68) or 120% of the anticipated days of intervention. There was a gradual progression in the difficulty of the opportunities parents documented providing. Three of the 5 infants progressed through all activities while 2 infants continued to work on a stage 1 activity. Parents retained the activity booklet and were asked to continue the activities until the end of phase 2 outcome visit.

### Primary outcomes

#### Reaching skill

Infants in both groups increased the duration they were in contact with the toy during the reaching trials with increasing age. (F = 5.33, *p* = 0.02) There was no significant Group-Time interaction (F_2,16_ = 0.32, *p* = 0.73) and no group differences in the duration of toy contact. However, infant in the SPEEDI group were in contact with the toy for a mean of 28.02 (16.3) out of 30 s in comparison to the usual care group 20.2 (21.45) seconds, 1 months after the intervention ended. Thus, the SPEEDI group approached a ceiling on this measure. The effect sizes for duration of toy contact were 0.11, 0.41, and 0.38 at endphase 2, followup 1 and followup 2 respectively suggesting a small but measureable effect of the intervention.

#### Exploratory problem-solving behaviors

**Fig. 2:**
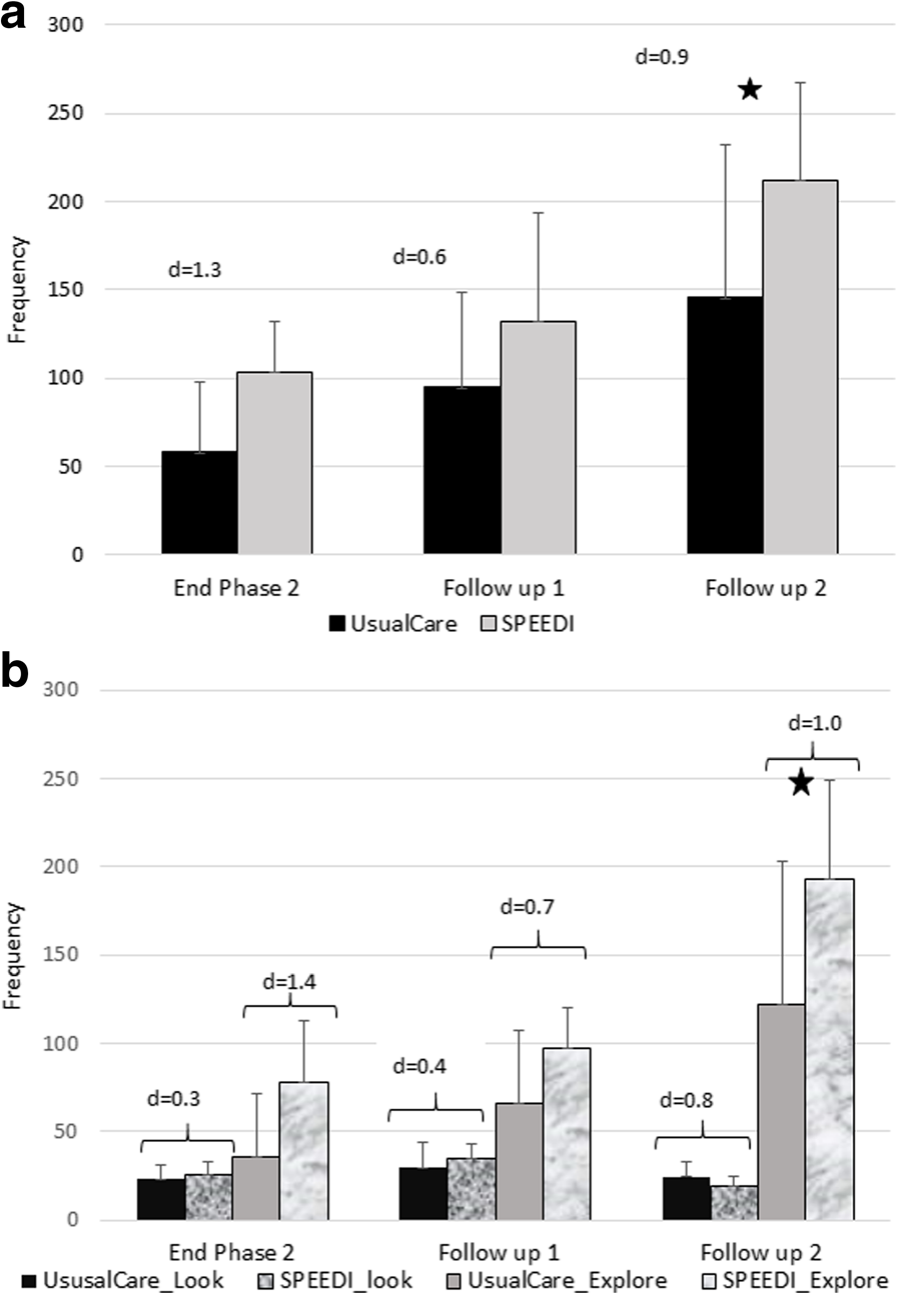
Problem Solving Outcomes. The frequency of problem solving behaviors during a 6-min interaction with 3 standardized toys. A: total problem solving behaviors. B: frequency of looks and explores, 2 specific types of problem solving behaviors. Star represented statistically significant group differences. Error bar represent 1 standard deviation from the mean. The effect size (d) for each comparison is included

Early problem solving behaviors increased in frequency with age in both groups (Fig. 2a and b). There is no significant group-time interaction for the sum of all early problem solving behaviors. However there was a significant group (F_1,8_ = 4.04, *p* = 0.08) and time effect (F_2,17_ = 9.76, *p* < 0.01, Fig. 2a). The Cohen D effects size for total problem solving behaviors and explore at the end of the intervention and during follow up were moderate to large (range 0.6 to 1.4, Figure 2a and b).

### Secondary outcomes

#### Neuromotor control and development

**Fig. 3:**
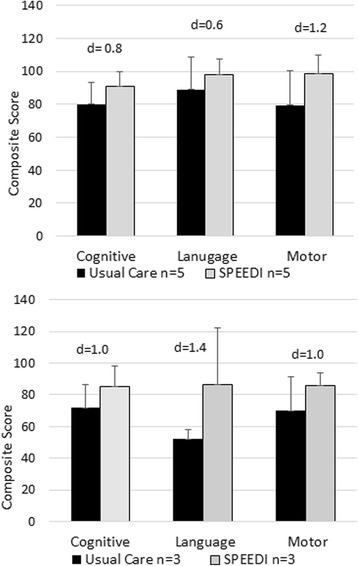
Global Development Outcomes. The Bayley composite score 3 months post intervention and at 12 months of age, approximately 9 months post intervention are provided for the Cognitive, Language (expressive and receptive), and Motor (Gross and Fine) domains. The 9 month post intervention visits includes infants who attended the Neonatal Continuing Care Program clinic visit and had a completed Bayley. Two infant in the SPEEDI group and 1 in the usual care group did not attend the clinic visit. One infant in the usual care group attended the clinic but could not complete the Bayley due to significant motor impairments

TIMP change in raw scores from baseline to the end of the intervention had a large effect size (d = 1.04). Longer-term global development outcomes on the Bayley had moderate to large effect sizes approximately 9 months post intervention at the 12 month adjusted age clinical assessment visit (Fig. 3a and b).

## Discussion

This initial efficacy randomized clinical trial suggest that intervention, such as SPEEDI, empowering parents to implement a daily routine of parent supported movement opportunities and environmental enrichment, has the potential to enhance development, even after the intervention has ended. Recent rehabilitation research on the treatment of children with motor impairments has emphasized the need for task specific and self-initiated movements to enhance learning [12, 40–42]. Parents of infants in the SPEEDI group were encouraged to identify ideal times to interact, set up the environment to provide a “just right challenge,” and support their infants self-initiated movements through a variety of activities. Based on parental adherence during collaborative parent, therapists, and infant sessions during phase 2 and the parent’s activities logs, the parents were able to utilize this training and incorporate these principles into their daily routine.

Infants adapt their arm and hand movements weeks before the onset of reaching [43]. While infant in both group increased their contact with toys during the reaching trials, infants in the SPEEDI group appear to have hit a plateau limiting the ability to quantify group differences. However, moderate to large effect sizes for the TIMP and the motor composite of the Bayley suggest that infants in the SPEEDI group had motor outcome scores higher than the usual care group, which might be statistically different with a larger sample size or with additional assessment of reaching earlier in the study period.

The majority of cognitive or problem solving assessments in infants and children required and are influenced by a child’s motor function [44, 45]. Likewise, a child’s ability to learn through interaction with the world can be influenced by motor impairments. All infants in this study improved their exploratory problem solving, primarily their exploration of objects, over the 3 months following the end of the intervention. However, the large effect sizes at all assessments, and statistically significant difference at follow up 2, suggest that infants in the SPEEDI group were able to demonstrate a higher frequency of exploratory problem solving behavior than the infants in the usual care group. While there is a requirement for motor activity to “explore” on the EPSI that may have contributed to the improved scores, the infants in the SPEEDI group appear to have higher cognitive scores at 3 and 9 months after the intervention reflected by the large effect sizes on the Bayley, supporting these initial efficacy finding on the EPSI.

While the results of this study are not conclusive and further study is needed, the SPEEDI intervention is consistent with current motor learning and developmental theory increasing the likelihood these findings are not extraneous. SPEEDI focuses around a few central tasks including support for infant initiated midline head and arm control, reaching, and object exploration in supine, sidelying, and prone. When the intervention started, most infants were unable to perform any of these tasks independently. However, the infants in this study had been moving in the extra uterine world for up to 16 weeks before starting this study. While not assessed in this study, interventions like SPEEDI may provide opportunities for activity dependent neuroplasticity to enhance the retention of the corticospinal fibers in infants with brain injury or immaturity and limit negative plasticity associated with a lack of variable movements [11]. In combination with supporting parents ability to provide daily opportunities’ to their infant, SPEEDI used a motor learning approach to increase repetitions of self-initiated movements that would not be possible in these infants without the environmental enrichment and support provided by the therapists or caregivers.

This initial evidence for the efficacy of SPEEDI challenges the current “wait and see” approach to early intervention and the medical community [9, 46]. SPEEDI is a feasible intervention if NICUs and state and federally supported early intervention program work together to ensure parents are given adequate information on the importance of providing an enriched environment, appropriately timed interactions, and support to enhance variable self-initiated movements. This cannot be done through a single session or generalized intervention strategies [47]. Parents appear to benefit from ongoing help to develop routines during the transition from the NICU to home that may lead to a decrease in the need for future services.

### Limitations

**Fig. 4:**
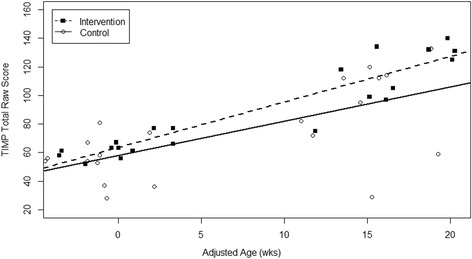
Group Differences in Motor Development with Increasing Age. The individual scores on the TIMP and predicted regression lines from the post hoc MLM with a significant interaction term. Suggests the rate of development was impacted by changes in age and group assignment

As a pilot and first efficacy study of this intervention, we planned to use an α = 0.10 for the primary outcome measures without correction of multiple comparisons in post-hoc testing. This limited our ability to conclude definitively on the efficacy of this intervention. The sample size was smaller than initially intended due to the loss of 4 enrolled infants. The inclusion of infant with significant brain injury and chronic lung disease resulted in 2 medical status changes that could not have been anticipated. These combined with the 2 voluntary drop outs reduced our sample to lower than the 7 infants per group needed to meet our planned power. We have included the effect sizes for the outcome assessments to enhance the readers’ ability to interpret the results with this small sample size. In addition, the loss of 2 infants with brain injuries eliminated our ability to do any type of sub-analysis to look at the efficacy of SPEEDI for infants with and without brain injury. Thus further data is needed on the efficacy of SPEEDI for infants at the highest risk of having CP. The planned use of reaching as a primary outcome, when the infants in the SPEEDI group reached a plateau limited our ability to fully describe group differences on the primary outcome measures. Infants in the SPEEDI group were more medically fragile resulting in an older gestational age before initiating intervention. Thus, it is possible that the group differences are not the result of the intervention, but are related to the older age of these infants at each data point. We addressed this where possible by evaluating change scores and plan to statistically control for age in future studies. In an attempt to evaluate the initial efficacy of SPEEDI controlling for age at assessment, we did a post-hoc analysis of the TIMP raw scores using a MLM including group, adjusted age at assessment, and an interaction term. The TIMP was our only measure that could be assessed from baseline to 1 month post intervention and thus was selected as the optimal measure for this post-hoc analysis. The interaction term was significant (F_1,29_ = 3.24, *p* = 0.08), Fig. 4. Using the predicted model from the MLM infants in the SPEEDI group gained 16.9 point more than the control group from baseline to 1 month post intervention (*p* = 0.07). This further supports the initial efficacy of SPEEDI, but requires additional research due to the preliminary and post-hoc nature of this analysis. While parent’s impressions of this intervention were not systematically collected in this study, they were in the feasibility study. Parents in the feasibility study reports that completing the activities daily was hard immediately post discharge but helped it become part of their routine interaction within a few weeks [33]. Additional qualitative study of group differences in parental impressions of the interventions would be beneficial in future studies.

Future research is need on the efficacy of SPEEDI to impact long term developmental outcomes in infant born very preterm, the need for future rehabilitation services, and quantification of changes in parent child interactions. A larger study of SPEEDI, including a comparison of the efficacy of SPEEDI for infant at the highest risk of CP, is in development and is needed before the efficacy of SPEEDI can be fully described.

## Conclusions

SPEEDI appears to have some benefit for infant born very preterm contributing to exploratory problem solving skills in the first months of life. Further research is needed, but preliminary evidence is promising, on the impact of SPEEDI on motor outcomes in infancy.

## Additional files

Additional file 1: Guiding Principles for SPEEDI Intervention. Includes the theoretical model and list of key principles of the Supporting Play Exploration and Early Development Intervention (SPEEDI). (DOCX 74 kb)

Additional file 2: List of Videos SPEEDI Phase 1. Lists the names and length of the videos provided to parents in SPEEDI Phase 1. These videos were available to the parents on an ipad or laptop computer for use during the 21 days of Phase 1 intervention. Parents were asked to watch all the videos at least 1 time, but had access to watch them as often as they wanted. (DOCX 16 kb)

Additional file 3: SPEEDI Activity Booklet. Includes the text from the SPEEDI activity booklet provided to parents toward the end of phase 1, for implementation in phase 2. Parents used the activity log in this appendix to document which activities were completed each day during Phase 2 of the SPEEDI intervention. (DOCX 19 kb)

## Abbreviations

CP: Cerebral palsy
EI: Early intervention governed by the US individuals with disability educational improvement act
EMR: Electronic medical record
EPSI: Early problem solving indicator
MLM: Mixed linear model
NICU: Neonatal intensive care unit
RMANOVA: A repeated measures analysis of variance
SPEEDI: Supporting play exploration and early development intervention
TIMP: Test of infant motor performance

## Footnotes

1 Datavyu 1.2, 196 Mercer St., 8th Floor, Suite 807, New York, NY 10012
