# Supplementary material for: Supporting play exploration and early developmental intervention versus usual care to enhance development outcomes during the transition from the neonatal intensive care unit to home: a pilot randomized controlled trial
Source: BMC Pediatr. 2018 Feb 9;18:46. doi: 10.1186/s12887-018-1011-4 (PMC5809115; doi:10.1186/s12887-018-1011-4)
Supplement: Supplementary file 1 — Guiding Principles for SPEEDI Intervention. Includes the theoretical model and list of key principles of the Supporting Play Exploration and Early Development Intervention (SPEEDI). (DOCX 74 kb) [file 12887_2018_1011_MOESM1_ESM.docx]

Additional file 1. Guiding Principles for SPEEDI Intervention

**Theoretical Model for SPEEDI**

The figure represents the 2 phases of the intervention (Phase 1 Blue and Phase 2 Red). The role of the parent and therapist are highlighted. The following guidelines were used in training and were the principles therapists utilized for the duration of intervention. In addition, the key principles listed below were taught to caregivers to guide their activities with their child during the parent delivered intervention sessions and general caregiving.


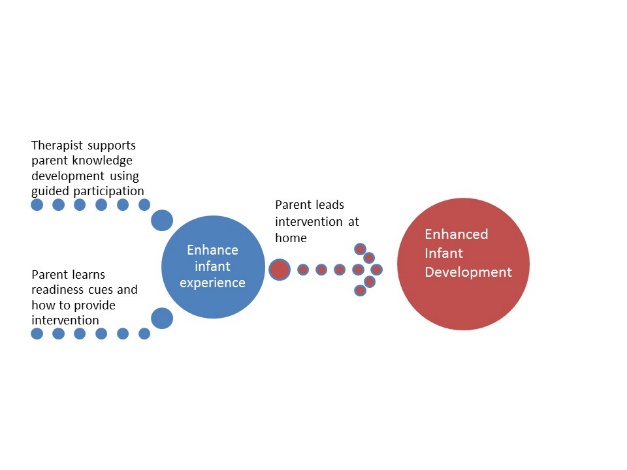


**SPEEDI Key Principles**

1. Identify alert and active times for intervention and follow the infants lead on when to provided rest breaks.
2. Develop a routine for when you will see the infant and how you engage with the infant.
3. Encourage participants to be active movers and explorers. This means you should give them ample time to move, make errors, and to correct their errors as independently as possible. It also means that when they require your assistance to transition between postures, to maintain postures, or to interact with objects, you should focus on providing them the least amount of assistance required.
4. Provide a “just right” challenge for participants. In order for participants to make continual gains in a safe, timely, and positive manner, it is important to know how much assistance they need to perform an activity and to understand ways you can adapt the activity to make it just challenging enough that the child will need to work harder but can achieve success. This can involve altering the level of assistance you provide, changing the child’s posture, or adapting aspects of the task such as object placement.
5. Encourage socialization and object interactions as a means to engage the infant. In order to elicit best performance from participants it will be necessary to understand their interests, likes, and dislikes. You will use this information to determine the objects you will utilize and ways you can structure tasks and rewards. Interaction does not need to be high level to be meaningful. However brief, periods of looking or toy contact are essential to providing meaningful experiences.
6. Development is nonlinear, and there are individual differences between children so that each child may take a slightly different path to the same endpoint. Do not have a “correct pattern” of movement in mind for early reaching or postural control; rather, let the child’s strategy emerge. The exploration of strategies is important in training a new skill.
7. Small and varied exploratory movements are essential building blocks for any new postural skill. Ample opportunities to explore diverse movements are necessary to support the development of postural control. These may include movements that you did not have in mind.
8. Errors are made with these exploratory and divergent trials, but these are valuable as long as they lead eventually to some successes in reaching an environmental goal.
9. Postural control can be affected by multiple factors including environment, sensation, perception, selective muscle control, practice, variability of that practice, cognition, overall health and temperament. Therefore you will need to keep in mind the interaction of the child with many factors.
10. Postural control is primarily prospective (as opposed to reactive), and used to engage with the environment; thus, intervention should focus on infant directed tasks, so prospective control is inherent in the therapeutic plan.
