# Supplementary material for: Supporting play exploration and early developmental intervention versus usual care to enhance development outcomes during the transition from the neonatal intensive care unit to home: a pilot randomized controlled trial
Source: BMC Pediatr. 2018 Feb 9;18:46. doi: 10.1186/s12887-018-1011-4 (PMC5809115; doi:10.1186/s12887-018-1011-4)
Supplement: Supplementary file 2 — List of Videos SPEEDI Phase 1. Lists the names and length of the videos provided to parents in SPEEDI Phase 1. These videos were available to the parents on an ipad or laptop computer for use during the 21 days of Phase 1 intervention. Parents were asked to watch all the videos at least 1 time, but had access to watch them as often as they wanted. (DOCX 16 kb) [file 12887_2018_1011_MOESM2_ESM.docx]

Additional file 2: List of Videos SPEEDI Phase 1

**Videos provided to parents for unlimited viewing during SPEEDI Phase 1**

1. Baby Demonstrating Readiness to Interact with a Caregiver – Behaviors we DO want (about 3.5 minutes)
   1. Readiness for Socialization– Behaviors we DO want

1a. Video file: Face to face socializing (33 seconds)

- 1. Readiness for Feeding– Behaviors we DO want

1b1. Video file: Feeding Readiness intro (1) (44 seconds)

1b2. Video file: Feeding Readiness quiet alert (2) (12 seconds)

1b3. Video file: Feeding Readiness rooting (3) (45 second)

1b4. Video file: Feeding Readiness searches for nipple (4) (20 seconds)

- 1. Readiness for Play– Behaviors we DO want

1c1. Video file: Hand to mouth (6 seconds)

1c2. Video file: ready to look at toys (31 seconds)

1. Baby demonstrating stability during activity – Behaviors we DO want (about 2.5 minutes)

2.1 Video file: Coordinated Infant Stability during feeding (40 seconds)

2.2 Video file: Tolerance to feeding (25 seconds)

2.3 Video file: Drowsy maintains muscle tone (25 seconds)

2.4 Video file: Quiet alert maintains muscle tone (32 seconds)

2.5 Video file: Learning head control (28 seconds)

1. Baby Demonstrating Lack of Readiness for Interaction – Behaviors we DO NOT want (6 minutes)

3.1 Video file: Social Disengagement (30 seconds)

3.2 Video file: Uncoordinated feeding readiness 2 intro (2.26 minutes)

3.3 Video file: low body tone (49 seconds)

3.4 Video file: unable to awaken does not root (42 seconds)

3.5 Video file: signs your baby needs are break (90 seconds)

3.6 Video file: Not ready for play (33 seconds)

1. Baby Demonstrating a Lack of stability during activity - Behaviors we DO NOT want (2.5 minutes)

4.1 Video file: Uncoordinated Stability (41 seconds)

4.2 Video file: Feeding Tolerance Uncoordinated (28 seconds)

4.3 Video file: Behavioral distress Grimace and avoid (37 seconds)

4.4 Video file: Arching away (35 seconds)

1. You and your baby are not ready to interact – DO NOT do this with your baby (2 minutes)

5.1 Video file: mother uncoordinated feeding behaviors (60 seconds)

5.2 Video file: Maternal uncoordinated feeding contact (15 seconds)

5.3 Video file: reduce distractions turn off the TV (18 seconds)

5.4 Video file: Social Disengagement (30 seconds)

1. Ways to help your baby be Ready to Interact – DO these with your baby (5 minutes)

6.1 Video file: Swaddle and hold (17 seconds)

6.2 Video file: Swaddling your infant (30 seconds)

6.3 Video file: Supportive Talk 2 (60 seconds)

6.4 Video file: Feeding Environment (43 seconds)

6.5 Video file: Cuddle and caress (56 seconds)

6.6 Video file: Maternal feeding behaviors (57 seconds)

6.7 Video file: Swaddle with low lights for play (41 seconds)
