# Supplementary material for: Supporting play exploration and early developmental intervention versus usual care to enhance development outcomes during the transition from the neonatal intensive care unit to home: a pilot randomized controlled trial
Source: BMC Pediatr. 2018 Feb 9;18:46. doi: 10.1186/s12887-018-1011-4 (PMC5809115; doi:10.1186/s12887-018-1011-4)
Supplement: Supplementary file 3 — SPEEDI Activity Booklet. Includes the text from the SPEEDI activity booklet provided to parents toward the end of phase 1, for implementation in phase 2. Parents used the activity log in this appendix to document which activities were completed each day during Phase 2 of the SPEEDI intervention. (DOCX 19 kb) [file 12887_2018_1011_MOESM3_ESM.docx]

Additional file 3: SPEEDI Activity Booklet

### SPEEDI Instructions for Home Play

Please direct questions to _____________________________

- Please perform the home play for a total of at least **20 minutes each day.** You can do this all at one time if your baby is awake and alert or break it up into shorter periods. Use your baby’s cues to help you decide. The goal is to make this play part of your daily routine and fun for you and your baby.
- Play should be performed when your baby is awake and alert but not crying.
- Establish a routine for when you will play. For example for 5 minutes after daytime diaper changes or for 10 minutes in the morning and 10 minutes in the afternoon. This will help you remember and help your baby learn to anticipate and enjoy this play time.
- The goal of the play is to help your baby get stronger, learn how to control his/her body, and learn about the world through play. You will help your baby learn to move his/her arms and legs, control his/her head, reach, and grasp toys by doing these activities when your baby is showing you signs he/she is alert and ready to play.
- Each activity has stage 1 and stage 2 activities. Most babies will do stage 1 activities for the first months the infant is in the study and at home. Once your baby can easily do the tasks in stage 1 you can try stage 2. The study team will help you decide when your baby is ready for stage 2 during the home visits.
- Each day after play, please take a minute to complete the daily journal sheet included in this booklet. Place a check in the box for any activity you did that day.
  - If you were unable to play that day, please note this by putting an X over that date’s entire page in the journal.
- You can make this play time more fun for you and your baby by talking or singing with your baby during the activities or you can incorporate other family members into the play activities. Follow your baby’s cue and limit activities if your infant shows signs of stress or has trouble interacting. Reduce activity and give your baby a break.
- Keep the room calm when you play so your baby can concentrate and so you can focus on your baby. (TV and music should be off, a quiet room without a lot of other activity is ideal)

**Signs I am ready to play:**

Picture were included here but can not be published

Sign I am **NOT** ready to play

Picture were included here but can not be published

PLAY ACTIVITIES FOR SPEEDI (pictures removed for publication)

(**20 minutes** total per day)

***Activity 1: Watching people and toys (4 minute)***

- - Stage 1-
    1. Hold your baby in your lap looking at you. Talk quietly to your baby to encourage your baby to open his/her eyes
    2. Move your head side to side to encourage your infant to follow you
    3. Show your baby a toy about 10-12 inches from him. Move it slowly to encourage him/her to look at it.
    4. If your baby has trouble with this, try wrapping/swaddling your baby or helping your baby keep his hands near his chest.
  - Stage 2 – Once your infant can follow a toy and your face in your lap…
    1. Place your infant on his back, side, or tummy to talk to your baby or show him/her toys to encourage him to look at them.
    2. Move the toys slowly so your baby can follow it.
    3. Allow the toys to bump into your baby’s hands sometimes.
    4. Encourage your infant to reach for the toy if interested.

***Activity 2: Tummy time (4 minutes).***

- - **Stage 1 –**

1. Place your baby on your chest and stomach or lap with his/her elbows under his/her shoulders.
2. Talk to your baby to encourage and entertain him/her to keep his/her head raised.
   - **Stage 2 – Once your baby can lift his/her head for at least 10 seconds on your chest**
     1. Place your baby on the floor on his/her stomach propped on his/her elbows to encourage use of the shoulder and head muscles.
     2. Hold a toy, mirror, or your face where your baby can see it and encourage your baby to look at the toy/face. Give him/her a few seconds to view it.
     3. Move the toy to the right and to the left. Encourage your baby to rotate his/her head to follow the toy.
     4. You can continue to do this on your chest lying flat on the floor if your baby prefers.
     5. If your baby has trouble lifting his/her head, try placing a hand on your baby’s bottom and give some gentle pressure and wait a few seconds to see if he/she can lift the head higher. Practice some of the time with this help and some without.
     6. Encourage your baby to stay on his/her stomach and lift his/her head for as long as possible. Take a break when needed then continue for a total of at least **4 minutes total with your baby being on his/her stomach**. You should build up to longer and longer time periods without a break.

***Activity 3: Holding Head Up (4 minutes).***

**Stage 1**

- 1. Hold your baby upright at your shoulder while you walk around or sway. Initially you may need to keep a hand on your baby’s upper back.
  2. Gradually move your hand so it is on your baby’s lower back and he/she can practice controlling his/her head more.

**Stage 2: - Once your baby can lift his/her head off your shoulder for 5 seconds**

- - 1. Support your infant in a sitting position with your hands around your baby’s torso AND arms so he/she can practice lifting his/her head. Only do this for 5-20 seconds. Talk with your baby and encourage your baby to look at you. Stop if your baby seems out of breath while doing this or is unable to lift the head at all and go back to stage 1.
    2. Once your baby can hold his/her head up for 20-30 seconds face your baby away from you so you can show your baby toys.

***Activity 4 : Kicking Play (4 minutes).***

**Stage 1:**

- 1. Place your baby on the floor, in your lap, or in a boppy pillow on his/her back. You can swaddle your baby’s arms if your baby gets upset being flat.
  2. Place your hands under your baby’s bottom or use a towel roll to bring his legs off the ground.
  3. Let your baby move his or her legs with this little bit of help and see what your baby does. We are looking for any kind of kicking or lifting of the legs off the surface.
  4. Kissing your baby’s feet and talking with them will make this fun.

**Stage 2: - Once your baby is moving his/her legs off the ground without help**

1. Put the wrist rattles on the ankles to see if your baby can see them.
2. Hang toys over your baby’s feet so they will accidentally bump them. Eventually this will help him/her learn to kick on purpose to hit the toys.

***Activity 5: Toy play with hands and legs in the middle (4 minutes).***

**Stage 1:**

- 1. Help your baby learn to bring his/her hands together and feet together in the middle of his body by making it a little easier to start with.
  2. Using a boppy pillow or your hands under your baby’s shoulder will naturally guide your baby’s hands toward each other.
  3. Your baby can play on his/her side so gravity can help bring the hands and feet near each other.
  4. Place the wrist rattles on your infant; place a toy in your baby’s hands and/or help your baby get hands to mouth.

**Stage 2:** Once your baby is starting to put his/her hands in her mouth, increase the amount of time your baby tries this activity without the help of the boppy or your hands. Continue to place the wrist rattle on wrists or ankles and place toys in your baby’s hands.

D**aily Journal: Play Day 1 Date:**

Was today a typical day for your infant: Yes No

If not please explain____________________________________________

Make a check mark () beside the activities that were completed today

| Activity | Stage 1  (easier, for younger babies) | Stage 2  (harder, for older babies) |
| --- | --- | --- |
| Activity 1: Watching people and toys |  |  |
| Activity 2: Tummy time |  |  |
| Activity 3: Holding Head Up |  |  |
| Activity 4: Kicking |  |  |
| Activity 5: Toy play |  |  |

Questions or problem to review at the next therapist meeting: __________________________

______________________________________________________________________________________________________________________________________________________
